# Prediction of cervical lymph node metastasis in solitary papillary thyroid carcinoma based on ultrasound radiomics analysis

**DOI:** 10.3389/fonc.2024.1291767

**Published:** 2024-01-25

**Authors:** Mei hua Li, Long Liu, Lian Feng, Li jun Zheng, Qin mei Xu, Yin juan Zhang, Fu rong Zhang, Lin na Feng

**Affiliations:** ^1^ Department of Ultrasound, Sijing Hospital of Songjiang District, Shanghai, China; ^2^ Department of Ultrasound, Shanghai General Hospital, Shanghai Jiao Tong University School of Medicine, Shanghai, China

**Keywords:** thyroid carcinoma, ultrasound, radiomics, lymph node metastasis, prediction

## Abstract

**Objective:**

To assess the utility of predictive models using ultrasound radiomic features to predict cervical lymph node metastasis (CLNM) in solitary papillary thyroid carcinoma (PTC) patients.

**Methods:**

A total of 570 PTC patients were included (456 patients in the training set and 114 in the testing set). Pyradiomics was employed to extract radiomic features from preoperative ultrasound images. After dimensionality reduction and meticulous selection, we developed radiomics models using various machine learning algorithms. Univariate and multivariate logistic regressions were conducted to identify independent risk factors for CLNM. We established clinical models using these risk factors. Finally, we integrated radiomic and clinical models to create a combined nomogram. We plotted ROC curves to assess diagnostic performance and used calibration curves to evaluate alignment between predicted and observed probabilities.

**Results:**

A total of 1561 radiomics features were extracted from preoperative ultrasound images. After dimensionality reduction and feature selection, 16 radiomics features were identified. Among radiomics models, the logistic regression (LR) model exhibited higher predictive efficiency. Univariate and multivariate logistic regression results revealed that patient age, tumor size, gender, suspicious cervical lymph node metastasis, and capsule contact were independent predictors of CLNM (all *P* < 0.05). By constructing a clinical model, the LR model demonstrated favorable diagnostic performance. The combined model showed superior diagnostic efficacy, with an AUC of 0.758 (95% CI: 0.712-0.803) in the training set and 0.759 (95% CI: 0.669-0.849) in the testing set. In the training dataset, the AUC value of the nomogram was higher than that of the clinical and radiomics models (*P* = 0.027 and 0.002, respectively). In the testing dataset, the AUC value of the nomogram model was also greater than that of the radiomics models (*P* = 0.012). However, there was no significant statistical difference between the nomogram and the clinical model (*P* = 0.928). The calibration curve indicated a good fit of the combined model.

**Conclusion:**

Ultrasound radiomics technology offers a quantitative and objective method for predicting CLNM in PTC patients. Nonetheless, the clinical indicators persists as irreplaceable.

## Introduction

Thyroid cancer stands as the most prevalent malignancy within the endocrine system. Among its histological variations, papillary thyroid carcinoma (PTC) takes precedence, accounting for approximately 90% of reported thyroid cancer cases. The prognosis for most PTC patients is quite favorable, with an impressive 10-year survival rate reaching up to 98%. However, in PTC patients, cervical lymph node metastasis (CLNM) is common, and it closely correlates with postoperative disease recurrence and survival prognosis (1, 2). Accurately predicting CLNM in preoperatively holds significant clinical value as it provides crucial guidance for selecting appropriate clinical treatment strategies.

The utilization of preoperative CT examination has been a common approach for assessing the presence of CLNM in PTC patients (3). However, its diagnostic sensitivity remains limited, especially in detecting subclinical lymph node metastasis, with a sensitivity of only 60%. Additionally, it is associated with relatively modest specificity and raises concerns about radiation exposure (4, 5). The primary benefit of preoperative CT lies in its ability to provide detailed insights into the dimensions, location, and characteristics of the primary thyroid tumor. Notably, it also facilitates the assessment of extrathyroidal extension (ETE), aiding in determining the extent of surgical resection and guiding appropriate surgical interventions (6). Currently, ultrasound examination serves as the primary method for preoperatively diagnosing CLNM in PTC patients. Conventional ultrasound (CUS) examination can determine the presence of CLNM by systematically scanning the cervical lymph nodes according to anatomical regions, and assessing changes in echogenicity, internal components, calcification, and Color Doppler flow imaging (CDFI) patterns of the cervical lymph nodes (7). However, it’s important to acknowledge that the CUS exhibits relatively lower sensitivity in diagnosing central compartment CLNM, with its diagnostic effectiveness primarily focused on detecting lateral CLNM (8, 9). Previous studies have explored the feasibility of predicting CLNM based on ultrasound characteristics and clinical features of the tumor in PTC patients. Nevertheless, predictive models constructed solely on preoperative clinical and ultrasound parameters tend to have limited effectiveness (10).

Radiomics is a quantitative method for medical imaging that aims to uncover tumor patterns and characteristics imperceptible to the naked eye by automatically extracting latent data features from medical images, thus providing important value for the precise diagnosis and treatment of tumors (11). Previous studies have shown that preoperative CT and MRI radiomics have significant value in assessing CLNM in PTC patients (12, 13). However, there have been relatively fewer studies on the role of ultrasound radiomics in evaluating CLNM in PTC patients (14). The objective of this study is to examine and confirm the effectiveness of different predictive models incorporating ultrasound radiomics features in predicting CLNM among patients diagnosed with solitary PTC.

## Materials and methods

### Patients

**Figure 1:**
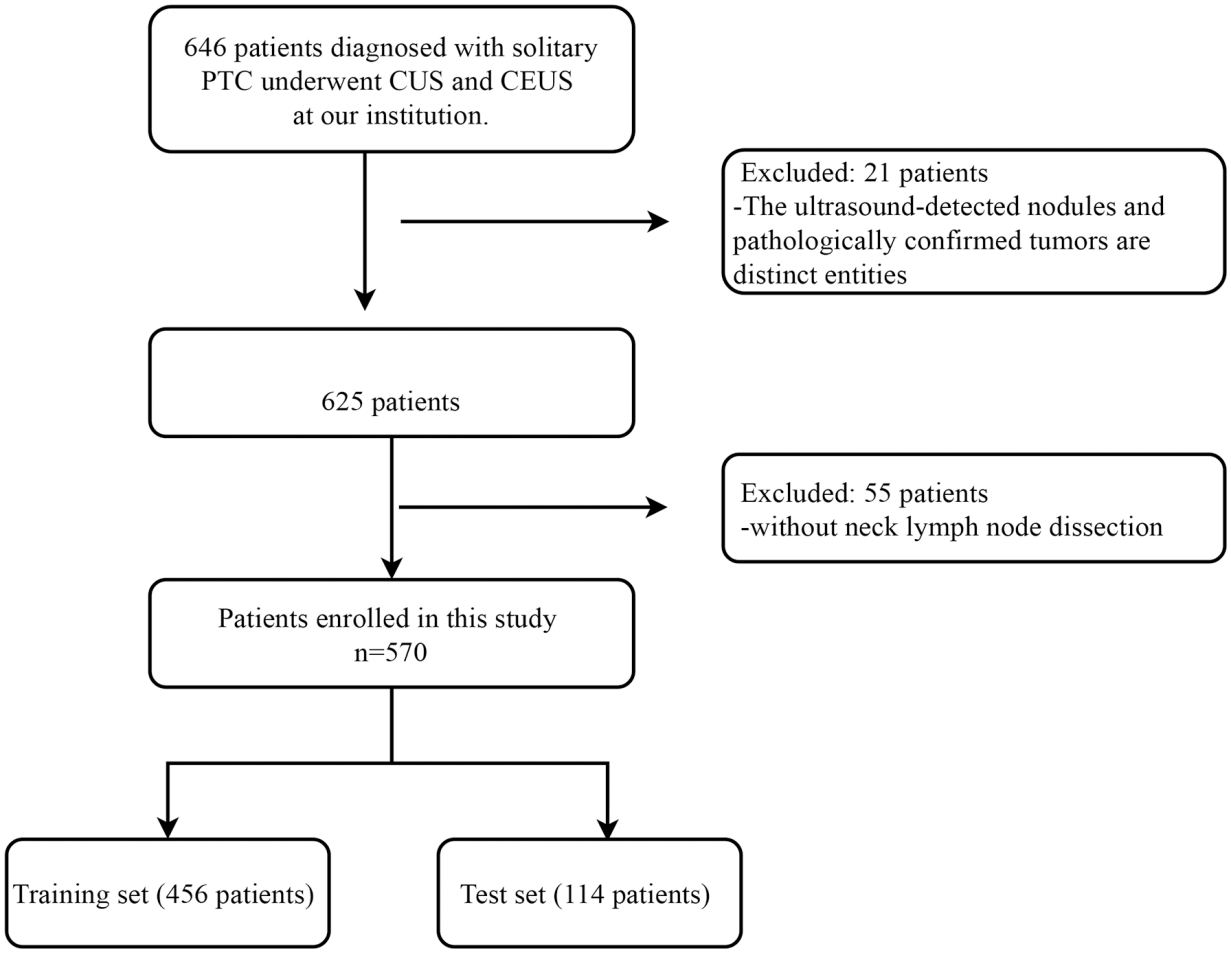
Patient inclusion and exclusion flowchart. This flowchart delineates the comprehensive patient inclusion and exclusion criteria for this study conducted on individuals diagnosed with solitary PTC at our institution. PTC, Papillary Thyroid Carcinoma; CUS, Conventional Ultrasound; CEUS, Contrast-enhanced Ultrasound.

With approval from our institutional ethics committee, we conducted a retrospective study. Due to the retrospective nature of the study, patients were exempted from the obligation of signing informed consent. The study population consisted of patients who underwent the CUS and contrast-enhanced ultrasound (CEUS) examinations at our hospital between January 2017 and December 2022 and were subsequently confirmed to have PTC on surgical pathology. The inclusion criteria were as follows: (1) patients who underwent preoperative CUS and CEUS at our hospital; (2) patients who eventually underwent surgery and were pathologically confirmed to have PTC; (3) patients with solitary nodules on both CUS and CEUS examinations. The exclusion criteria were: (1) mismatch between the nodule on ultrasound and pathology examinations; (2) patients who did not undergo cervical lymph node dissection. The flowchart of patient enrollment is shown in **Figure 1**. Finally, a total of 570 patients met the inclusion criteria of the study. Among them, there were 148 male and 422 female patients, with a mean age of 43.66 ± 11.90 years (range 17-80 years); the mean nodule size was 9.13 ± 6.01 mm (range 2.37-49.20 mm). According to the timing of recruitment, all patients were divided into training set (January 2017 - May 2020) and testing set (June 2021 - December 2022), with 456 patients in the training set and 114 patients in the testing set.

### Ultrasound examination

All thyroid ultrasound images were acquired using one ultrasound diagnostic systems, *Aplio 500* (Toshiba, Japan). During thyroid ultrasound examinations, we used high-frequency linear array transducers, with a frequency range of 7-14 MHz. To ensure clear image quality, we set the gain at 84dB and adjusted the depth and time-gain compensation appropriately. Patients were in the supine position with neck hyperextended and slightly rotated to one side, keeping the neck straight. Examinations started with CUS first, followed by CEUS. It is noteworthy that patients were required to sign informed consent before CEUS examinations.

In CUS, we first performed grayscale ultrasonography to scan the entire thyroid. During this process, we paid close attention to the thyroid’s size, echogenicity, and presence of nodules or masses. Upon detection of a concerning nodule, more detailed ultrasonographic characterization was undertaken, documenting the nodule’s location, number, size, echogenicity, margins, shape, presence of calcifications, relationship with the capsule, and assessment of capsule integrity. CDFI was also utilized to evaluate the vascularity within and surrounding the nodule. In addition to examining the thyroid and nodules, we also performed comprehensive ultrasound scans of the entire neck region to look for suspicious cervical lymph nodes metastasis (SLCNM). Lymph nodes were assessed for size, echogenicity, hilum, presence of calcifications, cystic degeneration, and vascular flow patterns. We stored the following static images of thyroid and cervical lymph node characteristics, including longitudinal and transverse grayscale images displaying the maximum diameters of nodules or lymph nodes, CDFI images, and images with typical imaging features such as liquefaction and calcification. Furthermore, we also stored the following dynamic images, including those depicting the nodule’s relationship with the thyroid capsule in transverse view, as well as images indicating the continuity of the thyroid capsule.

After the CUS examination, CEUS mode was activated. We used a low mechanical index of 0.001for the CEUS examination. We utilized dual imaging mode to simultaneously display the tumor location and the CEUS pattern. An intravenous catheter (20G) was inserted into the patient’s elbow vein, and 2 ml of contrast agent suspension was used. The ultrasound contrast agent was SonoVue (Bracco, Italy). The contrast agent was administered to the patient via bolus injection, followed by flushing with 10 ml of normal saline. Upon contrast injection, we started the timer on the ultrasound machine and recorded a video to document the dynamic contrast perfusion process for 1 minute.

### Ultrasound image and clinical data analysis

The ultrasound imaging features included tumor location, size, margin, shape, aspect ratio, calcification pattern, capsule contact, loss of capsule continuity, CDFI pattern, SCLNM, perfusion rate, homogeneity, enhanced intensity, and discontinuous capsule enhancement. The ultrasound imaging indicators are explained in detail in the **Supplementary Material**. All ultrasound images were independently assessed by two senior ultrasound specialists, each with over 5 years of experience in thyroid CEUS examinations. They were blinded to the CLNM features of PTC in the patients. The final assessments for each indicator were reached through consensus between these two assessors. In cases where there was disagreement between them, a third highly experienced physician with over 10 years of experience in thyroid CUS examinations reviewed the patient’s images, and the results were based on the assessment of the third physician.

Clinical data were retrieved from the hospital information system of our institution. The clinical data included patient’s age, gender, Hashimoto’s thyroiditis, surgical procedures performed, postoperative pathology results, and presence of CLNM. The solitary PTC was defined as the condition observed after the surgical treatment of a thyroid nodule. The conclusive pathological result verified the existence of a PTC focus within the patient’s thyroid, with only one such focus identified.

### Ultrasound image segmentation

**Figure 2:**
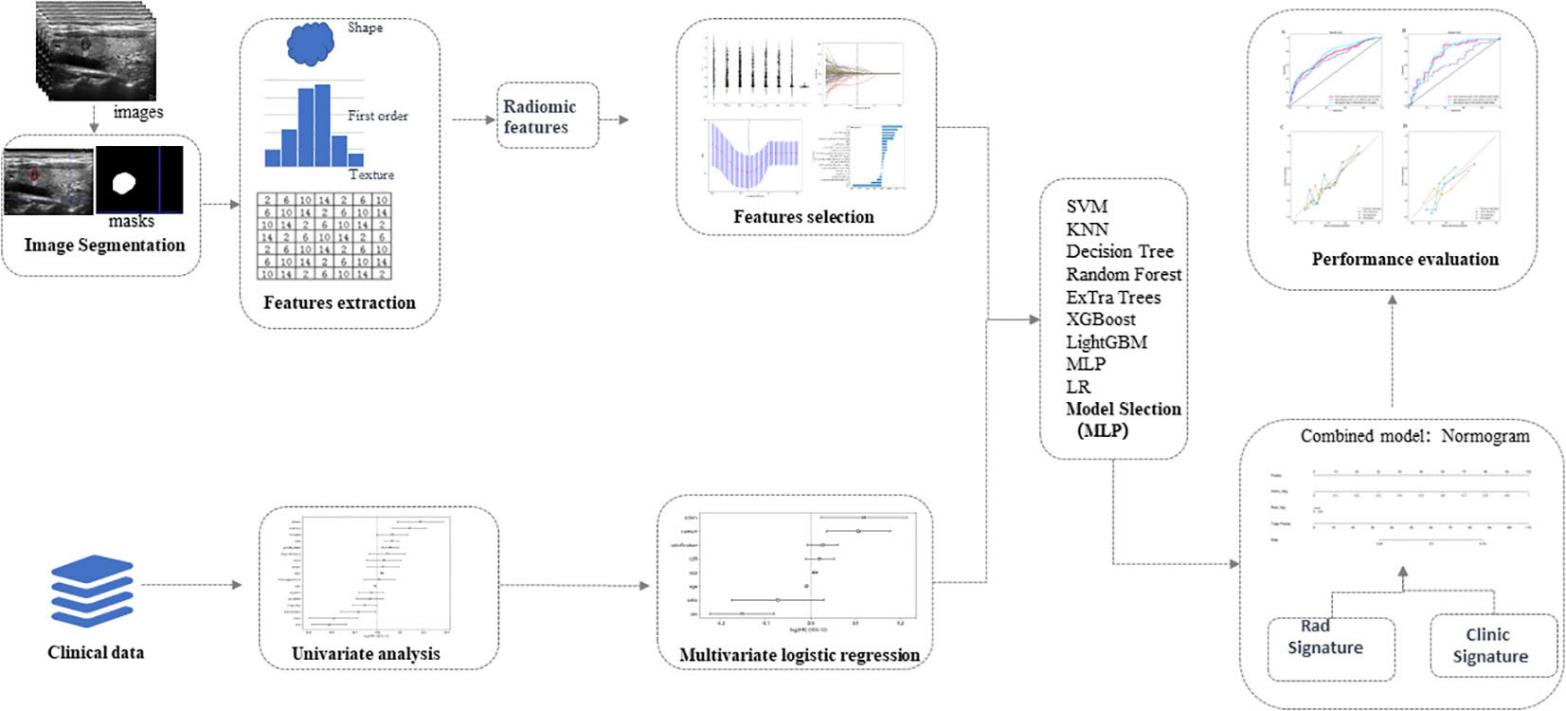
Schematic diagram of ultrasound radiomics analysis and model development. The radiomics approach is employed to extract features within the region of interest of thyroid tumors in ultrasound images. Valuable features are acquired through dimensionality reduction for machine learning to establish radiomics models. Univariate and multivariate analyses are conducted to investigate independent risk factors for cervical lymph node metastasis in papillary thyroid carcinoma, and clinical models are developed using machine learning techniques. Subsequently, a nomogram is generated based on the optimal results obtained from the radiomics and clinical models. The diagnostic performances of diverse models are thoroughly assessed and compared. LR, Logistic Regression; SVM, Support Vector Machine; KNN, K-Nearest Neighbors; RF, Random Forest; MLP, Multilayer Perceptron.

The workflow of radiomics analysis was shown in **Figure 2**. For radiomic analysis, grayscale ultrasound images (maximum diameter of tumor on longitudinal view) were acquired as jpg format before the region of interest (ROI) delineation and converted to nii.gz format. Ultrasound image preprocessing included resampling and normalization. Two radiologists with 5 and 7 years of experience in thyroid ultrasound examinations, blinded to CLNM status, utilized the ITK-SNAP software (version3.8.0, http://www.itksnap.org/) to delineate tumor ROIs from the images. The delineation was performed tightly along tumor margins. To ensure reliability and consistency of radiomics features, we randomly selected ultrasound images from 50 patients after one month and had the first radiologist re-delineate the ROIs. ICC (intraclass correlation coefficient) was used to assess intra- and inter-observer consistency. Parameters with ICC greater than 0.9 were considered to have good consistency and were included in the radiomics feature analysis. Radiomics features were extracted using the pyradiomics software (http://pyradiomics.readthedocs.io). These features can be categorized into three groups: (I) shape features, (II) intensity features, and (III) texture features. Shape features describe the three-dimensional shape characteristics of the tumor. Intensity features describe first-order statistical distributions of voxel intensities within the tumor. Texture features describe patterns in intensity, encompassing second- and higher-order spatial distributions of intensities. For texture feature extraction, various methods were utilized, including gray level co-occurrence matrix, gray level run length matrix, gray level size zone matrix, and neighborhood gray-tone difference matrix and gray level dependence matrix. After feature extraction, all radiomics features were normalized. Then, feature selection was performed using t-test or Mann-Whitney U test, retaining only radiomics features with p-value < 0.05. Subsequently, Spearman’s rank correlation coefficients were computed between features, and only one of features with correlation greater than 0.9 between any two was kept. To reduce feature dimensionality while maintaining descriptive capability, least absolute shrinkage and selection operator (LASSO) was employed. Through 10-fold cross-validation, the λ value that minimized cross-validation error was chosen, and the final retained non-zero coefficient features were used for model building. Next, a linear combination of the retained features was constructed, and a radiomics score was generated for each patient based on their model coefficients. All feature selection steps were performed on the training set, and the resultant features applied to the test set.

## Radiomics signature establishment

The Establishment of the radiomics model involved the following steps: First, after Lasso feature selection, we input the selected features into different machine learning (ML) models including logistic regression (LR), support vector machine (SVM), random forest, K nearest neighbors (KNN), ExtraTrees, XGBoost, LightGBM and multilayer perceptron (MLP) to build models for predicting the risk of CLNM. We used 10-fold cross-validation to derive the final radiomics signature. In evaluating model performance, we compared the diagnostic efficacy of the various models using metrics like area under the receiver operating characteristic curve (AUC), accuracy, sensitivity, specificity, positive predictive value (PPV) and negative predictive value (NPV). These metrics were used to determine the optimal radiomics model.

## Construction of clinical signature

For constructing the clinical model, we adopted the following approach: First, we performed univariate logistic regression analysis on clinical features, followed by multivariate logistic regression analysis on features with statistical significance to obtain the final predictors for establishing the clinical signature. It is important to emphasize that in developing the clinical signature, we similarly utilized the same ML models as the radiomics signature to ensure consistency of methodology and techniques.

## Development of combined model

After integrating the radiomics signature with the clinical signature, we established a combined model and visualized it as a nomogram. We selected the optimal model results for obtaining the combined model. By evaluating and validating on the training and test sets, we calculated a series of metrics including AUC, accuracy, sensitivity, specificity, PPV, and NPV to assess the model’s predictive performance. The AUC values are compared between the integrated model and the clinical and radiomics models in both the training and testing datasets. To assess the agreement between the models’ predicted outcomes and actual observations, we also plotted calibration curves.

## Statistical analysis

For statistical analysis, Python programming language (version 3.5.6) was used for data analysis. For continuous variables, mean and standard deviation or median and interquartile range were used for description, with t-test or U-test for intergroup comparison. For categorical variables, frequency or percentage was used for description, and chi-square test or Fisher’s exact test for analysis. For selection of clinical indicators, univariate and stepwise multivariate analyses were utilized. DeLong tests were employed to compare the AUC values among different models. A two-tailed *P* value of < 0.05 was considered statistically significant.

## Results

### Patients’ clinical and ultrasound imaging characteristics

This study included 570 patients (148 males and 422 females). The training set consisted of 456 patients, and the test set had 114 patients. The clinical and ultrasound characteristics of the patients are summarized in **Table 1**. Clinical features, such as age, size, aspect ratio, sex, location, SCLNM, shape, calcification, capsule contact, discontinuous capsule enhancement, CDFI, homogeneity, and Hashimoto’s thyroiditis were comparable between the training and testing sets (all *P* > 0.05). However, four features—margin, loss of capsule continuity, perfusion rate, and enhanced intensity—exhibit statistically significant differences between the training and testing sets (all *P* < 0.05). In the training dataset, age, size, aspect ratio, gender, presence of SCLNM, calcification pattern, capsule contact, CDFI, perfusion rate, and enhanced intensity exhibited significant differences between the CLNM (-) group and the CLNM (+) group (all *P* values < 0.05). In the testing dataset, age, size, calcification pattern, capsule contact, and the presence of Hashimoto’s thyroiditis showed significant differences between the CLNM (-) group and the CLNM (+) group (all *P* values < 0.05). Univariate and multivariate logistic regression analysis results showed that patient’s age (OR, 0.991; 95% CI, 0.988-0.994), size (OR, 1.009; 95% CI, 1.003-1.015), gender (OR, 0.859; 95% CI, 0.799-0.922), SCLNM (OR, 1.125; 95% CI, 1.023-1.239), and capsule contact (OR, 1.112; 95% CI, 1.036-1.194) were independent factors for predicting CLNM (**Table 2**).

**Table 1:** Analysis of clinical data and ultrasonographic features in training and testing sets of patients with PTC.

Features	Training set(n=456)			Testing set(n=114)		
	CLNM (-) (n=287)	CLNM (+) (n=169)	*P*	CLNM (-) (n=75)	CLNM (+) (n=39)	*P*
Age(year)	46.60 ± 11.71	39.55 ± 11.02	<0.001^*^	44.04 ± 10.65	39.13 ± 13.04	0.033^*^
Size(mm)	7.95 ± 4.19	10.56 ± 6.57	<0.001^*^	8.50 ± 6.91	12.84 ± 9.47	0.006^*^
Aspect ratio	1.15 ± 0.32	1.05 ± 0.28	0.002^*^	1.10 ± 0.32	1.08 ± 0.33	0.695
Sex			<0.001^*^			0.131
Male	52(18.12)	61(36.09)		19(25.33)	16(41.03)	
Female	235(81.88)	108(63.91)		56(74.67)	23(58.97)	
Location			0.553			0.398
Right	136(47.39)	89(52.66)		33(44.00)	16(41.03)	
Left	138(48.08)	73(43.20)		39(52.00)	23(58.97)	
Isthmus	13(4.53)	7(4.14)		3(4.00)	0 (0)	
SCLNM			0.002^*^			0.830
Absent	262(91.29)	137(81.07)		66(88.00)	33(84.62)	
Present	25(8.71)	32(18.93)		9(12.00)	6(15.38)	
Margin†			0.169			0.787
Clear	115(40.07)	56(33.14)		40(53.33)	19(48.72)	
Unclear	172(59.93)	113(66.86)		35(46.67)	20(51.28)	
Shape			0.66			1.000
Regular	104(36.24)	57(33.73)		32(42.67)	16(41.03)	
Unregular	183(63.76)	112(66.27)		43(57.33)	23(58.97)	
Calcification			<0.001^*^			0.033^*^
No	153(53.31)	67(39.64)		35(46.67)	9(23.08)	
Microcalcification	84(29.27)	73(43.20)		20(26.67)	20(51.28)	
Macrocalcification	38(13.24)	11(6.51)		11(14.67)	4(10.26)	
Mixed calcification	12(4.18)	18(10.65)		9(12.00)	6(15.38)	
Capsule Contact			0.038^*^			0.017^*^
Absent	92(32.06)	38(22.49)		27(36.00)	5(12.82)	
Present	195(67.94)	131(77.51)		48(64.00)	34(87.18)	
Loss of capsule continuity†			0.662			0.395
Absent	237(82.58)	136(80.47)		48(64.00)	21(53.85)	
Present	50(17.42)	33(19.53)		27(36.00)	18(46.15)	
Discontinuous capsule enhancement			0.524			1.000
Absent	213(74.22)	120(71.01)		55(73.33)	29(74.36)	
Present	74(25.78)	49(28.99)		20(26.67)	10(25.64)	
CDFI			0.014^*^			0.125
Type I	112(39.02)	47(27.81)		17(22.67)	9(23.08)	
Type II	35(12.20)	14(8.28)		15(20.00)	3(7.69)	
Type III	115(40.07)	84(49.70)		37(49.33)	19(48.72)	
Type IV	25(8.71)	24(14.20)		6(8.00)	8(20.51)	
Perfusion rate†			0.013^*^			0.229
Earlier	13(4.53)	19(11.24)		4(5.33)	5(12.82)	
Later	150(52.26)	73(43.20)		28(37.33)	10(25.64)	
Simultaneous	124(43.21)	77(45.56)		43(57.33)	24(61.54)	
Homogeneity			0.937			1.000
Homogeneous	140(48.78)	81(47.93)		35(46.67)	18(46.15)	
Heterogeneous	147(51.22)	88(52.07)		40(53.33)	21(53.85)	
Enhanced intensity†			0.018^*^			0.247
Hyper-enhancement	14(4.88)	20(11.83)		9(12.00)	9(23.08)	
Hypo-enhancement	119(41.46)	59(34.91)		26(34.67)	14(35.90)	
Iso-enhancement	154(53.66)	90(53.25)		40(53.33)	16(41.03)	
Hashimoto’s thyroiditis			0.552			0.017^*^
Absent	207(72.13)	127(75.15)		48(64.00)	34(87.18)	
Present	80(27.87)	42(24.85)		27(36.00)	5(12.82)	

^*^Indicates P<0.05 between the CLNM (-) and the CLNM (+) group.

† Indicates P<0.05 between the training set and the testing set.

SCLNM, suspicious cervical lymph node metastasis; CDFI, Color Doppler flow imaging.

**Table 2:** Univariate and multivariate analyses of clinical indicators.

Parameter	Univariate analysis			Multivariate analysis		
	OR	95%CI	*P*	OR	95%CI	*P*
Age(year)	0.989	0.986-0.992	<0.001^*^	0.991	0.988-0.994	<0.001^*^
Size(mm)	1.019	1.013-1.024	<0.001^*^	1.009	1.003-1.015	0.013^*^
Aspect ratio	0.828	0.744-0.921	0.004^*^	0.930	0.839-1.031	0.243
Sex	0.810	0.752-0.874	<0.001^*^	0.859	0.799-0.922	<0.001^*^
Location	0.966	0.912-1.024	0.331			
SCLNM	1.207	1.091-1.331	0.002^*^	1.125	1.023-1.239	0.041^*^
Margin	1.067	0.997-1.142	0.114			
Shape	1.025	0.957-1.099	0.556			
Calcification	1.058	1.020-1.096	0.011^*^	1.027	0.993-1.064	0.190
Contact	1.149	1.068-1.236	0.002^*^	1.112	1.036-1.194	0.014^*^
LCC	1.044	0.965-1.131	0.372			
DCE	1.208	0.954-1.108	0.535			
CDFI	1.065	1.033-1.100	0.001^*^	1.020	0.989-1.053	0.290
Perfusion rate	0.974	0.923-1.027	0.413			
Homogeneity	1.007	0.941-1.077	0.864			
Enhanced intensity	0.946	0.899-0.995	0.073			
HT	0.921	0.855-0.993	0.072			

^*^Indicates P<0.05.

LCC, Loss of capsule continuity; DCE, Discontinuous capsule enhancement; HT, Hashimoto’s thyroiditis; CDFI, Color Doppler Flow Imaging; SCLNM, Suspected Lymph Node Metastasis.

### Clinical prediction model results

Using the independent risk factors for CLNM, clinical signatures were built for the training and testing sets using ML models (**Table 3**). Results showed that RandomForest, ExtraTrees and XGBoost models exhibited overfitting. After comparative analysis, it was observed that the AUC values of LR and MLP models were similar on the training set. However, the AUC value of the LR model was higher than that of the MLP model on the testing set. Therefore, we ultimately chose the LR model as the optimal model, with AUC of 0.757, sensitivity of 0.897, specificity of 0.622, accuracy of 0.711, PPV of 0.547, and NPV of 0.920.

**Table 3:** Diagnostic performance of different clinical models in the training and test sets.

Models	AUC	95% CI	Acc.	Sen.	Spe.	PPV	NPV
LR							
Train	0.728	0.679-0.776	0.719	0.485	0.857	0.667	0.739
Test	0.757	0.666-0.849	0.711	0.897	0.622	0.547	0.920
SVM							
Train	0.691	0.639-0.743	0.691	0.521	0.808	0.615	0.741
Test	0.738	0.642-0.835	0.728	0.667	0.760	0.591	0.814
KNN							
Train	0.803	0.766-0.841	0.695	0.84	0.61	0.559	0.866
Test	0.646	0.540-0.752	0.693	0.487	0.822	0.559	0.75
RF							
Train	0.995	0.991-0.999	0.974	0.976	0.972	0.954	0.986
Test	0.659	0.557-0.761	0.632	0.564	0.685	0.468	0.746
ExTra Trees							
Train	1.000	1.000-1.000	0.996	1.000	0.993	0.988	1.000
Test	0.636	0.527-0.745	0.632	0.692	0.643	0.474	0.789
XGBoost							
Train	0.928	0.904-0.951	0.860	0.858	0.861	0.784	0.911
Test	0.684	0.579-0.789	0.623	0.821	0.520	0.471	0.848
LightGBM							
Train	0.825	0.788-0.863	0.743	0.805	0.707	0.618	0.860
Test	0.680	0.576-0.784	0.614	0.795	0.520	0.463	0.830
MLP							
Train	0.701	0.651-0.751	0.686	0.538	0.774	0.583	0.740
Test	0.702	0.596-0.808	0.684	0.744	0.653	0.527	0.831

LR, logistic regression; SVM, Support Vector Machine; KNN, K-Nearest Neighbors; RF, Random Forest; MLP, Multilayer Perceptron; Acc., accuracy; Sen., sensitivity; Spe., specificity; PPV, Positive Predictive Value; NPV, Negative Predictive Value.

### Ultrasound imaging radiomics prediction model results

**Figure 3:**
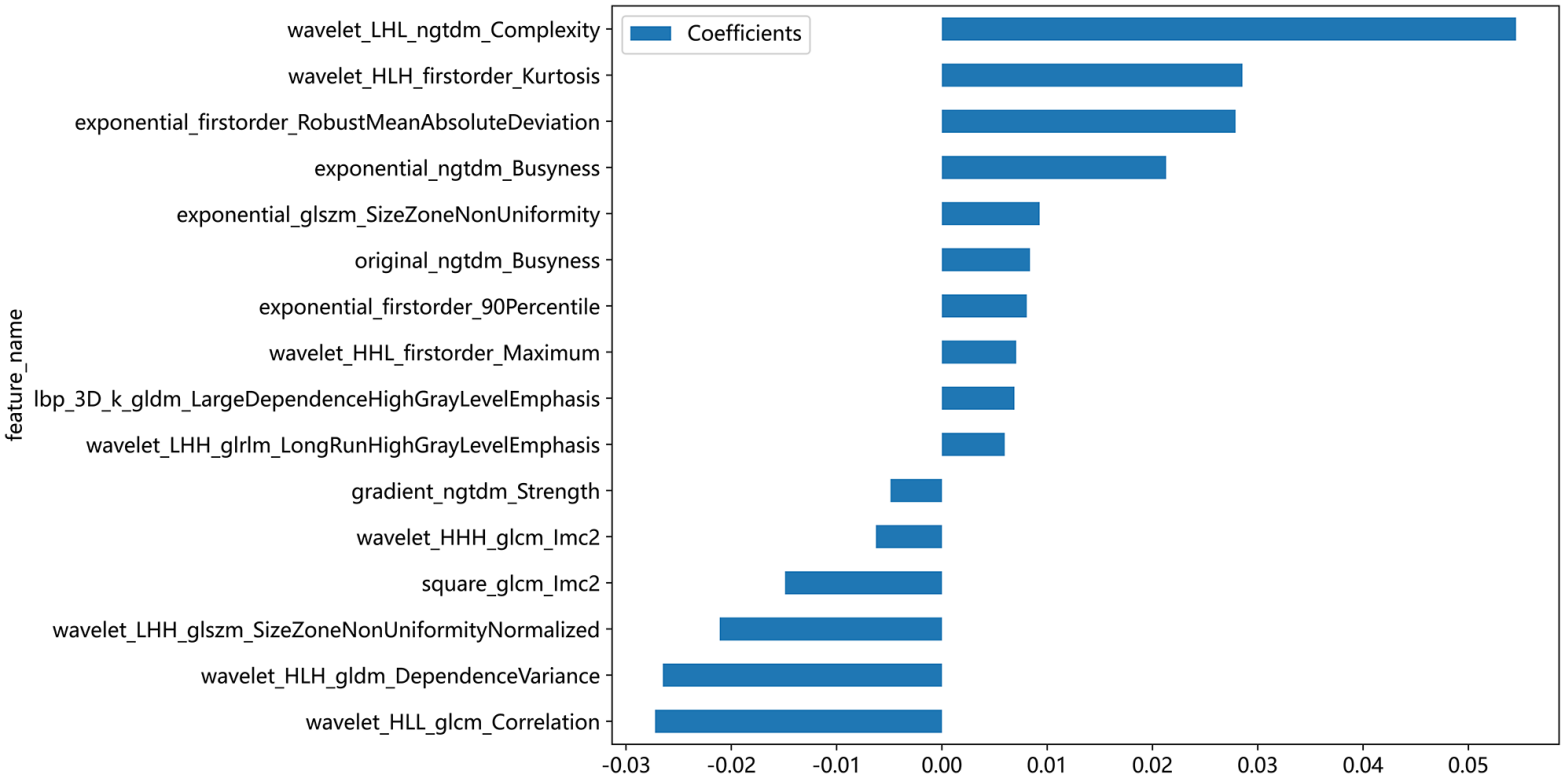
Coefficient values of selected ultrasonographic radiomics features for machine learning modeling. This bar chart illustrates the coefficient values of ultrasonographic radiomics features that were selected for inclusion in machine learning models. Each bar represents the magnitude of the feature’s coefficient, with positive values indicating a feature that contributes positively to the model’s output and negative values indicating a negative contribution.

A total of 1561 radiomics features were extracted from each patient’s ultrasound image. After feature selection, 16 non-zero features were finally chosen for ML modeling (**Figure 3**). Using ML approaches, 8 radiomics models were built for the training and test sets respectively (**Table 4**). Based on the AUC performance of different ML models on the training and testing sets, we evaluated that SVM, KNN, RandomForest, ExtraTrees, XGBoost, LightGBM models exhibited overfitting. It was observed that The AUC values of LR and MLP models were similar on both the training set. However, the AUC value of the LR model was higher than that of the MLP model on the testing set. Therefore, we ultimately chose the LR model as the optimal model with a sensitivity of 0.513, specificity of 0.720, accuracy of 0.649, a PPV of 0.488, and a NPV of 0.740.

**Table 4:** Diagnostic performance of different ultrasound radiomics models in the training and test sets.

Models	AUC	95% CI	Acc.	Sen.	Spe.	PPV	NPV
LR							
Train	0.701	0.651-0.750	0.656	0.645	0.662	0.529	0.76
Test	0.630	0.520-0.740	0.649	0.513	0.72	0.488	0.740
SVM							
Train	0.831	0.792-0.871	0.761	0.888	0.686	0.625	0.912
Test	0.581	0.463-0.700	0.675	0.462	0.787	0.529	0.737
KNN							
Train	0.796	0.758-0.835	0.689	0.858	0.589	0.551	0.876
Test	0.584	0.479-0.690	0.579	0.667	0.533	0.426	0.755
RF							
Train	0.998	0.996-1.000	0.991	0.988	0.993	0.988	0.993
Test	0.572	0.460-0.684	0.553	0.667	0.493	0.406	0.74
ExTra Trees							
Train	1.000	1.000-1.000	1.000	1.000	1.000	1.000	1.000
Test	0.565	0.456-0.675	0.482	0.821	0.307	0.381	0.767
XGBoost							
Train	0.995	0.990-0.999	0.963	0.976	0.955	0.927	0.986
Test	0.595	0.483-0.707	0.588	0.615	0.573	0.429	0.741
LightGBM							
Train	0.953	0.935-0.971	0.888	0.929	0.864	0.801	0.954
Test	0.561	0.446-0.676	0.684	0.256	0.919	0.588	0.701
MLP							
Train	0.737	0.689-0.784	0.686	0.663	0.700	0.566	0.779
Test	0.608	0.496-0.719	0.658	0.436	0.784	0.500	0.725

LR, logistic regression; SVM, Support Vector Machine; KNN, K-Nearest Neighbors; RF, Random Forest; MLP, Multilayer Perceptron; Acc., accuracy; Sen., sensitivity; Spe., specificity; PPV, Positive Predictive Value; NPV, Negative Predictive Value.

### Construction and evaluation of the combined model

**Figure 4:**
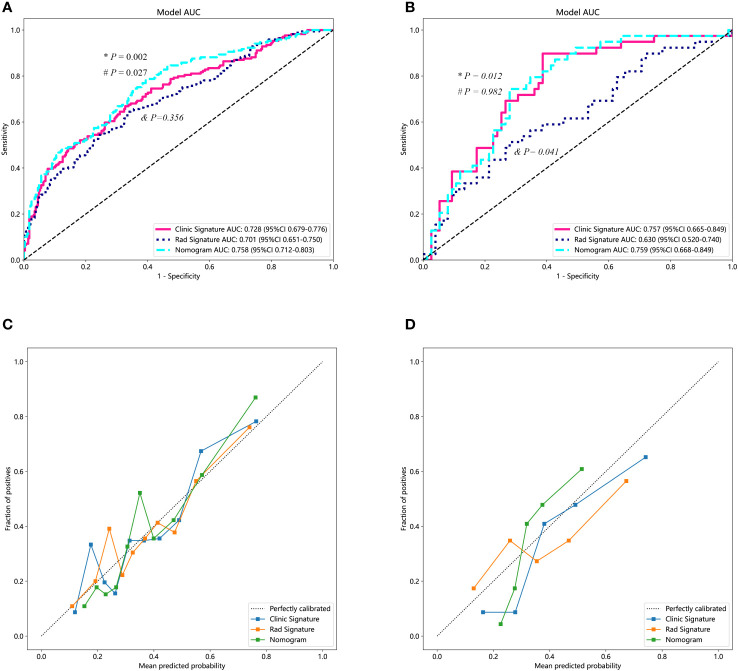
Diagnostic performance of different predicting models. **(A)** The performances of various models in the training set. The AUC value of the nomogram was superior to both the clinical and the radiomics models. Additionally, the clinical model demonstrated a similar AUC value compared to the radiomics model. **(B)** The performance of the models in the testing set. Both the nomogram and the clinical model exhibited higher AUC values than that of the radiomics model. **(C, D)** represent the calibration curves for the three models in the training and testing datasets, respectively. “*” represents the statistical *P*-value when comparing the AUCs of the nomogram and the radiomics model. “#” indicates the statistical *P*-value when comparing the AUCs of the nomogram and the clinical model. “&” indicates the statistical *P*-value when comparing the AUCs of the clinical model and the radiomics model. AUC, area under the curve.

We used the optimal model results to construct a combined model, which we then visualized as a nomogram. To ensure consistent and objective assessment of clinical utility of the models, LR was chosen for both the clinical and radiomics signatures. In the training dataset, the AUC value of the nomogram was greater than that of the clinical and radiomics models (*P* = 0.027 and 0.002, respectively). Moreover, there was no significant statistical difference observed in the AUC values between the clinical model and the radiomics model (*P* = 0.356). In the testing dataset, the AUC value of the nomogram model was also greater than that of the radiomics model (*P* = 0.012). However, the statistical difference between the nomogram and the clinical model was not significant (*P* = 0.928). Furthermore, the AUC of the clinical model was higher than that of the radiomics model (*P* = 0.041). The nomogram demonstrated good agreement between predicted and actual observed values in both training and testing sets (*P* values for Hosmer-Lemeshow test were 0.280 and 0.051, respectively) (**Figure 4**).

## Discussion

The findings of this study demonstrate that the radiomics model based on ultrasound image features has limited value in preoperatively predicting CLNM for patients with PTC. However, combining the radiomics features with clinical data can improve predictive performance. This result may offer valuable insights for tailoring personalized treatment strategies for PTC patients in clinical practice.

This study revealed an association between age and CLNM in PTC patients, indicating younger patients were more prone to developing CLNM. This is consistent with a recent study that also showed a negative correlation between age and CLNM, despite different age group cutoffs (15). Our study also revealed a higher CLNM detection rate in males, which is consist with the observations of Zhu et al. (16). The negative correlation in females may be related to hormonal and reproductive factors (17). Additionally, there was a positive correlation between larger tumor size and the CLNM. Larger tumors had a greater tendency to develop CLNM, consistent with prior research findings (18, 19). This study identified tumor contact with the capsule as an independent risk factor for CLNM. In agreement with Wang et al. (20), we also emphasized the association between tumor-capsule correlation and CLNM. Seong et al. found that a distance from the capsule <1.9 mm was associated with CLNM in PTC patients (21). Different from their quantitative method, we opted for the assessment of capsule contact as the indicator, aiming to facilitate a more practical clinical application of our prediction model. Moreover, consistent with previous studies, the preoperative detection of SCLNM on ultrasound was also identified as a risk factor for CLNM (22, 23). Despite the significant value of preoperative clinical characteristics in predicting CLNM, there are limitations in subjectivity for evaluation and analysis of certain ultrasound features. Hence, this study aimed to explore more objective, automated, and accurate preoperative CLNM prediction approaches to overcome these limitations.

Radiomics is a methodology that involves the extraction of a multitude of quantitative features from medical images through the utilization of data characterization algorithms. This method transforms digital medical images into high-dimensional data that is imperceptible to the human eye, extracting meaningful information hidden in the images that may have value for decision support, personalized medicine, and predictive modeling. This study highlights the substantial significance of texture features in predicting CLNM within the tumor region of PTC. Following a dimensionality reduction analysis of radiomics features extracted from the tumor’s ROI, a total of 16 radiomics features were ultimately retained and employed for machine learning modeling. Among these features, 75% (12/16)are texture features, concentrating on assessing the contrast of grayscale distribution, the consistency and repeatability of the texture, the complexity and disorder of the texture, as well as the linear correlation of grayscale values between pixels and their neighboring pixels in the tumor. Additionally, 25% (4/16) features are categorized as first-order features. These first-order features evaluate tumor heterogeneity by scrutinizing variations in pixel intensity within the tumor region. They encompass the mean, median, standard deviation, maximum, and minimum pixel intensities in the tumor, providing insights into the distribution and fundamental properties of pixel intensities in the image, including overall brightness, contrast, and uniformity. This finding was consistent with the results reported by Park et al. (24). These features delineated the intensity and distribution of gray levels within tumors, which could potentially be associated with alterations in the structure and density of tumor cells. Radiomics features can reflect underlying pathologic alterations, thereby providing important evidence for preoperative prediction of CLNM in PTC patients.

In this study, we performed ML modeling using radiomics features from ultrasound images after Lasso feature selection. However, logistic regression (LR) was eventually chosen as the model. While the majority of ML models, including SVM, KNN, random forest, ExtraTrees, XGBoost, and LightGBM, demonstrated strong performance on the training set, their AUCs exhibited a significant decline on the testing set, indicating overfitting problems. In comparison, the LR model maintained relatively high AUC on the testing set. Similarly, in building clinical signature, we also utilized various MLmodels, which showed overfitting issues with RandomForest, ExtraTrees, XGBoost, LightGBM models. Ultimately, we selected the model that demonstrated superior performance on the testing set as the clinical signature, which remained the LR method. In exploring the integration of clinical and radiomics models, we combined the optimal results of the clinical model with those of the radiomics model to create a combined model. Results demonstrated the combined model achieved the highest AUC, followed by the clinical model, and then the radiomics model. In this study, the AUC value of the radiomics model was similar to the clinical model in the training set but lower in the testing set. This suggests that, in clinical practice, radiomics technology cannot replace traditional clinical indicators. We also found that a new model combining radiomic and clinical outcomes showed higher diagnostic performance in both the training and testing sets compared to the standalone radiomics model. Additionally, the combined model exhibited superior performance only in the training set compared to the clinical model. In the testing set, their diagnostic performance was similar, indicating the crucial value of clinical indicators in predicting CLNM in PTC patients. However, one of the primary advantages inherent in radiomic method is their ability to provide an objective assessment (25). In clinical practice, it is essential to combine ultrasound radiomics features along with clinical characteristics to optimize the preoperative prediction of CLNM in patients with PTC.

There are some limitations in this study. First, this was a single-center retrospective study, which may introduce selection bias. Thus, the results need to be verified in larger-sample, multi-center, prospective studies. Second, evaluation of clinical ultrasound features has a certain degree of subjectivity. Although two experienced physicians performed independent assessments and consistency evaluation was carried out in this study, subjectivity may still affect the diagnostic performance of the clinical and combined models. Third, this study only analyzed radiomics features of the primary thyroid tumor ultrasound images, without in-depth analysis of radiomics features of the lymph nodes. In future studies, we plan to construct multi-modal combined models to incorporate lymph node radiomics features and thereby further improve model prediction performance. Finally, tumor ROI delineation in this study was performed manually, which is notably inefficient. In future studies, we will try automatic delineation approaches to reduce human intervention and improve research efficiency and objectivity.

## Conclusion

Ultrasound radiomics technology provides a quantitative and objective means for predicting CLNM in PTC patients. However, the value of traditional clinical indicators remains irreplaceable, underscoring the imperative need for their combined utilization in clinical practice.

## Data availability statement

The raw data supporting the conclusions of this article will be made available by the authors, without undue reservation.

## Ethics statement

The studies involving humans were approved by Medical Ethics Committee of Sijing Hospital in Songjiang District. The studies were conducted in accordance with the local legislation and institutional requirements. The ethics committee/institutional review board waived the requirement of written informed consent for participation from the participants or the participants’ legal guardians/next of kin because this was a retrospective study.

## Author contributions

ML: Conceptualization, Data curation, Formal analysis, Methodology, Software, Visualization, Writing – original draft, Writing – review & editing. LL: Methodology, Software, Writing – review & editing. LF: Data curation, Investigation, Writing – review & editing. LZ: Data curation, Investigation, Writing – review & editing. QX: Data curation, Investigation, Writing – review & editing. YZ: Investigation, Writing – review & editing. FZ: Data curation, Investigation, Writing – review & editing. LNF: Conceptualization, Supervision, Writing – review & editing.
