# Supplementary material for: Prediction of cervical lymph node metastasis in solitary papillary thyroid carcinoma based on ultrasound radiomics analysis
Source: Front Oncol. 2024 Jan 25;14:1291767. doi: 10.3389/fonc.2024.1291767 (PMC10850287; doi:10.3389/fonc.2024.1291767)
Supplement: Supplementary file 1 [file DataSheet_1.docx]

Supplementary Material

Prediction of Cervical Lymph Node Metastasis in Solitary Papillary Thyroid Carcinoma Based on Ultrasound Radiomics Analysis

First Author*, Co-Author, Co-Author

*** Correspondence:** Corresponding Author: email@uni.edu

# Ultrasound Image Analysis

# Tumor location can be classified as right lobe, left lobe, or isthmus. Tumor margin can be clear or unclear. Tumor shape refers to regular or unregular. Aspect ratio is the anteroposterior to transverse diameter ratio on the transverse section with the maximum diameter. Calcification types include no calcification, microcalcification, coarse calcification, and mixed calcification. Capsule contact is defined as whether the tumor periphery is in contact with the thyroid capsule, categorized as absent or present. Loss of capsule continuity refers to whether the continuity of the capsule high echogenic linear is interrupted on grayscale ultrasound, classified as absent or present. Based on color Doppler flow within the tumor and surrounding thyroid parenchyma (STP), four types can be identified: Type I, no obvious color flow signals are seen within the tumor and STP; Type II, abundant blood flow signals are detectable in the STP, but sparse flow signals within the tumor; Type III, sparse blood flow distribution is shown in both the tumor and STP; Type IV, abundant blood flow signals are seen within the tumor. Suspicious cervical lymph node metastasis is characterized by specific ultrasound features, including the absence of the fatty hilum, peripheral vascular flow on color Doppler imaging, elevated cortical echogenicity, a rounded shape (long axis to short axis ratio of <1.5), cystic changes, and microcalcification. Perfusion rate denotes the relative timing of contrast agent appearance between the tumor and STP during the contrast-enhanced ultrasound, categorized as earlier (tumor enhancement earlier than STP), later (tumor enhancement later than STP), and simultaneous (tumor and STP enhance simultaneously). Enhanced intensity indicates the relative enhancement degree within the tumor compared to the STP at peak contrast agent perfusion, classified as hyper-enhancement (higher intensity within tumor), hypo-enhancement (lower intensity within tumor), and iso-enhancement (similar intensity as STP). Homogeneity of tumor is categorized into homogeneous and heterogeneous enhancement. Discontinuous capsule enhancement refers to interrupted continuity of the highly enhanced capsular during contrast-enhanced ultrasound, defined as absent or present.
